# Designing of a multi-epitopes based vaccine against *Haemophilius parainfluenzae* and its validation through integrated computational approaches

**DOI:** 10.3389/fimmu.2024.1380732

**Published:** 2024-04-16

**Authors:** Sana Abdul Ghaffar, Haneen Tahir, Sher Muhammad, Muhammad Shahid, Tahir Naqqash, Muhammad Faisal, Thamer H. Albekairi, Abdulrahman Alshammari, Norah A. Albekairi, Irfan Manzoor

**Affiliations:** ^1^ Department of Bioinformatics and Biotechnology, Government College University, Faisalabad, Pakistan; ^2^ Institute of Molecular Biology and Biotechnology, Bahauddin Zakariya University, Multan, Pakistan; ^3^ Food Department, Government of Punjab, Lahore, Pakistan; ^4^ Department of Pharmacology and Toxicology, College of Pharmacy, King Saud University, Riyadh, Saudi Arabia; ^5^ Department of Biology, Indiana University, Bloomington, IN, United States

**Keywords:** LptD, BamA, *Haemophilius parainfluenzae*, subtractive proteomics, multi-epitopes

## Abstract

*Haemophilus parainfluenzae* is a Gram-negative opportunist pathogen within the mucus of the nose and mouth without significant symptoms and has an ability to cause various infections ranging from ear, eye, and sinus to pneumonia. A concerning development is the increasing resistance of *H. parainfluenzae* to beta-lactam antibiotics, with the potential to cause dental infections or abscesses. The principal objective of this investigation is to utilize bioinformatics and immuno-informatic methodologies in the development of a candidate multi-epitope Vaccine. The investigation focuses on identifying potential epitopes for both B cells (B lymphocytes) and T cells (helper T lymphocytes and cytotoxic T lymphocytes) based on high non-toxic and non-allergenic characteristics. The selection process involves identifying human leukocyte antigen alleles demonstrating strong associations with recognized antigenic and overlapping epitopes. Notably, the chosen alleles aim to provide coverage for 90% of the global population. Multi-epitope constructs were designed by using suitable linker sequences. To enhance the immunological potential, an adjuvant sequence was incorporated using the EAAAK linker. The final vaccine construct, comprising 344 amino acids, was achieved after the addition of adjuvants and linkers. This multi-epitope Vaccine demonstrates notable antigenicity and possesses favorable physiochemical characteristics. The three-dimensional conformation underwent modeling and refinement, validated through *in-silico* methods. Additionally, a protein-protein molecular docking analysis was conducted to predict effective binding poses between the multi-epitope Vaccine and the Toll-like receptor 4 protein. The Molecular Dynamics (MD) investigation of the docked TLR4-vaccine complex demonstrated consistent stability over the simulation period, primarily attributed to electrostatic energy. The docked complex displayed minimal deformation and enhanced rigidity in the motion of residues during the dynamic simulation. Furthermore, codon translational optimization and computational cloning was performed to ensure the reliability and proper expression of the multi-Epitope Vaccine. It is crucial to emphasize that despite these computational validations, experimental research in the laboratory is imperative to demonstrate the immunogenicity and protective efficacy of the developed vaccine. This would involve practical assessments to ascertain the real-world effectiveness of the multi-epitope Vaccine.

## Introduction

Despite tremendous advances in medical research, bacterial infections continue to be a major cause of death (1). Vaccination is a key part of public health efforts, helping control the spread of germs and significantly lowering mortality rates (2). However, it is essential to note that existing vaccines are not universally employed at 100% coverage on a global scale (3). Ongoing efforts are focused on the development of new vaccines, leveraging emerging technologies for antigen identification and formulation, coupled with our increasing understanding of mechanisms underlying immune responses to vaccination (4). Globally, vaccination programs are estimated to save between 2 to 3 million lives annually and substantially reduce the burden of disease for tens of millions of individuals. Furthermore, vaccination programs also lead to significant economic savings (5).

Gram-negative bacteria pose a significant worldwide health challenge mainly because they have a higher resistance to antibiotics (6). Antibiotic resistance creates significant hurdles in medical contexts, particularly within hospital environments, where patients in intensive care units (ICUs) are particularly vulnerable. Infections stemming from these microorganisms can result in severe illness and even fatalities (7). *Haemophilus parainfluenzae* is a gram negative bacterium belongs to the HACEK group of organisms (8). *H. parainfluenzae* is associated in a spectrum of clinical conditions, encompassing instances of brain abscess, epidural abscess, liver abscess, epiglottitis, and bacteremia. In young adults, it can cause subacute endocarditis (9). In adults, *it* can lead to more common illnesses such as upper respiratory infections, chest colds, and bronchial tube inflammation. The composition of *H. parainfluenzae* includes heat-adjustable proteins of around 37 kDa, peptidoglycan-associated proteins, and lipopolysaccharides (10).

The emergence of multidrug-resistant (MDR) phenotypes in various bacterial species is a consequence of genetic diversity and recombination. In case of the human-adapted bacterium, it is increasingly becoming an adaptable multidrug-resistant pathogen (11). In recent times, *in silico* approaches have garnered significant attention due to their potential to expedite drug discovery while reducing the associated time, labor, and costs (12). These *in silico* approaches have been successful in developing many new drug compounds (13). Computational methodologies in drug design are pivotal in drug discovery process, serving to efficiently identify potential drug candidates while managing costs effectively. To be more precise, *in silico* approaches prove valuable in the field of pharmacological research by diminishing dependence on animal models. They empower the rational design of innovative and safe drug candidates, providing crucial support to medicinal chemists at every stage of the drug discovery process (14). Computational vaccinology is also gaining prominence as it offers a solution to the challenges associated with vaccine design. Predicting B cell and T cell epitopes is an important step in the design and development of vaccines against many bacteria, viruses, and malignancies (15). This entails locating particular areas on antigens recognized by T cells and B cells, which are essential components of the immunological response. Analyzing the possibility that an antigen would trigger an immune response is known as antigenicity analysis (16).

The goal of this work is to use *in silico* methods to discover potential vaccine targets by connecting the *H. parainfluenzae* proteome and genetic data. This study looked at the proteome of *H. parainfluenzae* T3T1 to find potential candidates for vaccination. Epitopes on T and B cells have been predicted using proteins from *H. parainfluenzae*. Highly antigenic and conserved epitopes were selected first, and linkers and adjuvants were subsequently added to make subunit vaccines. The vaccine’s physicochemical properties, structural characteristics, allergenicity, and antigenicity were examined using online resources. Furthermore, docking was done between TLR-4 and the vaccine. *In-silico* cloning was the last technique employed to investigate the advanced polyprotein synthesis. Using a unique combination of several antigenic peptides produced by the bacterium, this research may result in the development of effective and dynamic immunizations that prevent infection with *H. parainfluenzae*.

## Materials and methods

### Protein sequence retrieval and conserved region identification

The entire proteome sequence data in FASTA format was acquired by downloading from UniProt, a freely accessible database providing information on protein sequences and functions (17). Their functional importance and pathogenicity based on their essential proteins. To attenuate the risk of triggering an autoimmune response, making sure the chosen proteins lacked homology with human proteins was essential (18). To achieve this, the BlastP bioinformatics tool was employed. BlastP is a widely used bioinformatics tool that conducts sequence similarity (19). This involved compare query sequence against a comprehensive database of protein sequence. Finding the similarities between query and database protein sequences is the primary goal of this procedure (20). Moreover, the VaxiJen server was employed to assess the antigenicity of the chosen proteins by applying a predetermined 0.05 threshold (21).

### CTL epitope prediction

Cytotoxic T lymphocyte (CTL) cells plays a vital part in the immune system by admit certain antigens (22). To identify CTL epitopes, the interested protein sequences were given that in FASTA format, CTLPred was used in MHC class I complex (23). Integrated epitope prediction was utilized, focusing on selecting CTL epitopes with consensus scores below 2. A lower score indicates an enhanced capability of these epitopes to bind to MHC-I molecules, making them favorable candidates for the construction of vaccine sequences (24). The purpose of CTLPred is to identify putative T cell epitopes in a particular protein sequence by emphasizing their capacity to bind to MHC-I molecules, which makes it easier for cytotoxic T cells to recognize them (25). We utilized the VaxiJen 2.0 server to evaluate the antigenicity of these chosen epitopes. When referring to an epitope’s ability to elicit an immune response, the term “antigenicity” is used (26). To predict allergenicity and toxicity, additional tools such as AllerTOP 2.0 and ToxinPred servers were utilized (27). This comprehensive approach ensures that the selected epitopes not only possess the potential to activate the immune system but are also non-allergic and non-toxic, making them promising candidates for vaccine development (28).

### HTL- epitope prediction

Helper T lymphocytes (HTL) play a crucial role as integral components within the adaptive immune system, demonstrating the capability to coordinate both cellular and antibody-mediated immune responses directed against foreign pathogens (29). In the context of vaccine development, epitopes on Helper T lymphocyte (HTL) cells that interact with MHC class II alleles are essential. These T cells collaborate synergistically with B cells to generate antibodies that specifically target and neutralize active pathogenic cells. This collaborative effort is integral as they work in conjunction with Cytotoxic T Lymphocytes (CTL) and macrophages to collectively combat and eliminate harmful pathogens (30).

### Prediction and evaluation of B-cell epitopes

B-cell epitopes play a pivotal role in initiating an adaptive immune response and serve as fundamental components in the design of vaccines (29). The ABCPred bioinformatics tool, which is adept at predicting antigenic epitopes or antibody-binding sites within protein sequences, was utilized in the current investigation to predict B-cell epitopes (31). The goal of ABCPred is to anticipate linear B-cell epitopes, or certain areas of proteins that are recognized and bound to by antibodies (32). A threshold value of 0.5 was employed in this tool to predict linear B-cell epitopes within the protein sequences. The VaxiJen 2.0, ToxinPred, and AllerTOP 2.0 servers were utilized to assess the antigenicity, toxicity, and allergenicity of the predicted B-cell epitopes (33). This thorough evaluation ensures that the predicted B-cell epitopes not only possess the potential to stimulate an immune response (antigenicity) but are also non-toxic and non-allergenic, rendering them suitable candidates for inclusion in vaccine constructs (34).

### MEV construction

By carefully integrating adjuvant and epitopes from Cytotoxic T-lymphocyte sources, Helper T-lymphocyte and B-cell the multi-epitope vaccine (MEV) sequence was constructed. Specific linkers were used to ensure effective functionality in the integrated design (35). Choosing an adjuvant to control the immunological response that the vaccine evoked was a crucial decision. In this particular case, the adjuvant chosen was the subunit B of cholera enterotoxin (36).

The construction of the MEV sequence followed a strategic approach:

1. Adjuvant Placement: The adjuvant, cholera enterotoxin subunit B, was strategically positioned at the N-terminal of the vaccine construct (36). The incorporation of the adjuvant at the N-terminal of the vaccine construct was facilitated by utilizing an EAAAK linker, ensuring the seamless integration of the adjuvant into the vaccine sequence (37).
2. Linkers for Epitope Separation: Efficient separation between each epitope is essential for their individual and effective functioning (38). Specific linkers were employed to achieve this separation:

- CTL Epitopes: The chosen CTL epitopes were linked together using AAY linkers (37).
- HTL Epitopes: Likewise, the HTL epitopes were connected using GPGPG linkers (39).
- B-cell Epitopes: The linear B-cell epitopes were connected together through the KK linker. This linker choice helps in preserving their distinct immune response (40).

### Structure analyzing

The MEV sequence underwent a series of thorough assessments and analyses to guarantee its safety and efficacy as a candidate vaccine (41): Homology Testing: To verify that the MEV sequence did not exhibit significant similarity to human proteins, a BlastP search against the Homo sapiens proteome was conducted with neglect framework (42). This step aimed to avoid potential autoimmune responses. Antigenicity and Immunogenicity Evaluation: The antigenicity and immunogenicity profiles of the MEV sequence were assessed through analysis using the VaxiJen and IEDB servers, respectively (43). The purpose of this phase was to assess the sequence’s ability to elicit an immunological response. Using the ProtParam service, the modified vaccine’s physicochemical characteristics were ascertained (44). These properties included:

- Molecular weight
- Instability index
- Theoretical isoelectric point (pI)
- Half-life in vivo and in vitro
- Aliphatic index (AI)
- GRAVY (Grand Average of Hydropathy) values (45)

These factors provide important information about the MEV sequence’s general properties, charge, and stability, contributing to the assessment of its potential as an effective vaccine (43). These parameters help assess the vaccine’s stability and suitability for practical applications (46). Toxicity and Allergenicity Prediction: To ensure the vaccine’s safety, its toxicity was assessed using the ToxinPred server, and its potential for inducing allergies was evaluated through the AllerTOP server (47). These analyses were conducted to confirm that the vaccine candidate was non-toxic and devoid of allergic responses (48).

### Tertiary structure prediction, refinement and validation

The three-dimensional (3D) structure of a protein is critical to its stability and function. Your vaccine’s tertiary structure was created using a multi-step process that guaranteed the protective structure’s accuracy and predicted its function: The initial vaccine 3D structure was generated using the Alpha- fold 2 server (49). GalaxyRefine Server: The projected 3D structure was further refined and enhanced through the GalaxyRefine server (50). This action was taken to improve the precision and excellence of the forecasted protein structure. RAMPAGE Server: To validate the refined structure, you used the RAMPAGE server (51). RAMPAGE assesses the quality of a protein structure by examining its stereochemical properties, such as bond angles and dihedral angles. This validation step helps ensure that the refined structure conforms to expected structural standards (52). ProSA-web Server: For structural verification ProSA server was used. The structural quality were find by using this server, the lower score have more valid structure (53).

### Prediction of discontinuous B-cell epitopes

The discontinuous B-cell epitopes were located using the ElliPro program (54). The tertiary structure was also determined using MEV. ElliPro uses the protein’s 3D structure as input to detect areas where amino acids conjoin to form epitope (24). The PI score is used for identifying discontinuous B-cell epitopes. Higher PI scores specify that the part of protein to be involved in antibody binding (55). This information is important for vaccine construct that might interact with antibodies and immune response (56).

### Disulfide engineering

By creating disulfide connections between cysteine residues, disulfide engineering seeks to improve the stability of a protein’s three-dimensional structure (57). These bonds are essential for maintaining the stability of the molecular interactions inside the protein (58). The improved model was presented in Design v2.0. This specialized server is designed to predict and recommend the placement of disulfide bonds within a protein structure. It achieves this by analyzing both the protein’s amino acid sequence and its structural information (59).

### Molecular docking with host immune receptor

Establishing an effective immunological response relies significantly on the interaction between a vaccine protein and host immune cells (60). To assess how well your MEV could bind to human immunological receptors, molecular docking research was conducted (61). One specific immunological receptor investigated in this research is Toll-like receptor 4 (TLR4) (62). The Toll-like Receptor 4 (TLR4) is pivotal in the human immune system as it recognizes and responds to foreign microbes with high sensitivity and specificity (63). It responds to molecular patterns associated with pathogens (PAMPs), such as lipooligosaccharides and lipopolysaccharides, which can activate TLR4-mediated immune responses (64).

### Normal mode analysis of MEV-receptor docked complex

Normal mode analysis is frequently included in *in silico* studies to assess the stability of protein-protein complexes (65). This assessment involves analyzing the dynamics of proteins and contrasting their behavior with their typical modes (66). One method to study protein mobility is through normal mode analysis (NMA), which assesses the common motions of proteins within their inherent coordinates (67). In my research, the iMODS server was employed for this purpose (68). The iMODS (NMA) server provides valuable insights into the intrinsic motions of a multiplex of proteins (69). It calculates various parameters, including eigenvalues, covariance, B-factors, and deformability, to characterize the protein’s dynamics and stability (70). Eigenvalues: These values indicate the primary chain’s deformability. Smaller eigenvalues suggest that the protein structure is more prone to bending or undergoing deformations (71). This property is directly associated with the energy required to induce such deformations. Covariance: Covariance analysis helps identify correlated motions within the protein structure, shedding light on how different parts of the protein move together (72). Higher B-factors indicate greater flexibility and mobility. The dynamics of the protein-protein complex under study can be explored by using the iMODS server for normal mode analysis (73). This information is crucial for assessing how the complex behaves and whether it is suitable for its intended function in the context of a vaccine or other biomedical application (74).

### Immune simulation

Through utilization of C-ImmSim, investigators have the capability to computationally simulate and forecast the immune reactions elicited by the MEV construct upon its introduction into the organism (75). This *in silico* methodology provides significant utility in evaluating the prospective efficacy of the vaccine and acquiring understanding of its immunological ramifications, circumventing the necessity for exhaustive *in vivo* or *in vitro* experimentation (76).

### Codon optimization and *in silico* cloning

The Java Codon Adaptation Tool (JCat) was employed in the study to carry out several essential tasks related to codon optimization and adaptation (77). The first step entails back translation, converting the designed vaccine’s amino acid sequence into a nucleotide (DNA) sequence (78). This process is known as back translation. The optimization make sure that the gene is expressed efficiently in the host organism (79). The CAI value is indicates that how well optimized codon matches the codon of host organism (80). A higher CAI value indicates a better match. The optimized sequence GC content is determined. This is important because the GC content of the host organism may change gene expression (81). The protein sequence of the vaccine was fed into the JCat server, and strain K12 of *E. coli* was selected as the host (82). Using SnapGene 3.2.1 software, the optimized sequence was included into the pET30a (+) expression vector following the codon optimization process (83). This vector is frequently used to ensure that the vaccine design is correctly translated and transcribed in the host organism by expressing recombinant proteins in *E. coli* (84).

## Results

### Proteome sequences retrieval

The entire proteome of *H. parainfluenzae (*strain T3T1) consists of 1974 proteins. By using the web server Geptop-2.0, 370 essential proteins inferred from these. By eliminating human homologous by an online analysis using BlastP, there were 180 non-homologous protein recognized. To assess these antigenicity values were used. The top two proteins with the highest antigenicity were composed of extracellular proteins, and they were subsequently selected for additional screening (**Table 1**).

**Table 1:** Detailed information about antigenic vaccine protein of *H. parainfluenzae*.

Accession no	Protein	Antigenicity	Allergenicity	Toxicity
|E1W2K9|	LPS-assembly protein LptD	0.5865	Non-allergen	Non -toxin
|E1W430|	Outer membrane protein assembly factor BamA	0.6128	Non -allergen	Non -toxin

### Epitope selection and evaluation of CTL

Based on the *H. parainfluenzae* target protein, 42 CTL epitopes 12-mer were prioritized. For the purpose of developing vaccines, the top seven epitopes exhibiting high immunogenicity and antigenicity as well as being non-toxic and non-allergenic were selected using a variety of technologies to evaluate antigenicity, immunogenicity, allergenicity, and toxicity (85) (**Table 2**). 37 unique HTL epitopes were identified, and the top four were chosen for vaccination based on their cytokine-induced capabilities (**Table 3**). Similarly, among the selected 19 LBL epitopes, two were deemed suitable for vaccine development, considering predictions related to toxicity, allergenicity, and antigenicity (**Table 4**).

**Table 2:** Final CTL selected epitopes for the construction of vaccine against *H. parainfluenzae*.

Epitope	Protein	Allele	Position	Antigenicity	Immunogenicity
ASTGVAFQW	Outer membrane protein assembly factor BamA	HLA-E*01:03	750-758	1.3793	0.15485
FDFSFGWNYNSL	Outer membrane protein assembly factor BamA	HLA-B*39:01 HLA-C*03:03 HLA-C*12:03	562-573	1.7566	0.12233
IYDNSIGVTFEL	LPS-assembly protein LptD	HLA-A*24:02 HLA-A*23:01	740-751	1.4082	0.23623
KSMNLTGNSIKT	Outer membrane protein assembly factor BamA	HLA-A*32:01	548-559	1.5931	-0.17922
NSIKTNDFDFSF	Outer membrane protein assembly factor BamA	HLA-A*32:01	555-566	1.7487	0.01632
RFNFSAGQIYYL	LPS-assembly protein LptD	HLA-A*30:02 HLA-A*29:02	572-583	1.1200	0.0329
YYQPNYNVAISA	LPS-assembly protein LptD	HLA-C*14:02 HLA-C*07:02 HLA-A*23:01	353-364	1.0204	0.02773

**Table 3:** Final HTL epitopes for the construction of vaccine against *H.parainfluenzae*.

Epitope	Protein	Allele	Position	Antigenicity	Immunogenicity
QPNYNVAISARQFQI	LPS-assembly protein LptD	HLA-DRB1*09:01	355-369	1.2124	0.04526
YQPNYNVAISARQFQ	LPS-assembly protein LptD	HLA-DRB1*09:01	354-368	1.0527	0.08425
FKVLGVPVFYTPYLQ	LPS-assembly protein LptD	HLA-DRB1*11:28 HLA-DRB1*13:05	197-211	0.5298	0.233
RFKVLGVPVFYTPYL	LPS-assembly protein LptD	HLA-DRB1*11:28 HLA-DRB1*13:05	196-210	0.5329	0.19876

**Table 4:** Final selected B-cell epitope for the construction of vaccine.

Epitope	Protein	Score	Position	Antigenicity	Immunogenicity
TKLYATHYNQKKGSSS	LPS-assembly protein LptD	0.77	516	1.3164	-0.647
YETYDNSKSDTSSTYK	Outer membrane protein assembly factor BamA	0.8	1422	1.2321	-0.73485

### Construction of multi-epitope chimeric vaccine

All of the chosen epitopes were included in the building of the vaccine; the MHC-I, LBL, and MHC-II epitopes were linked together using AAY, KK, and GPGPG linkers, respectively (86). The presentation of the epitope and immunization are improved by these linkers (39). The first CTL epitopes were added as an adjuvant, coupled with cholera enterotoxin component B. These epitopes were utilized to inhibit the generation of junctional epitopes, facilitated by the incorporation of the EAAAK linker. Linkers made of EAAAK increase structural stability (87). The final 344 amino acid vaccination demonstrates the arrangement of different epitopes and linkers.

### Population coverage estimation

**Figure 1:**
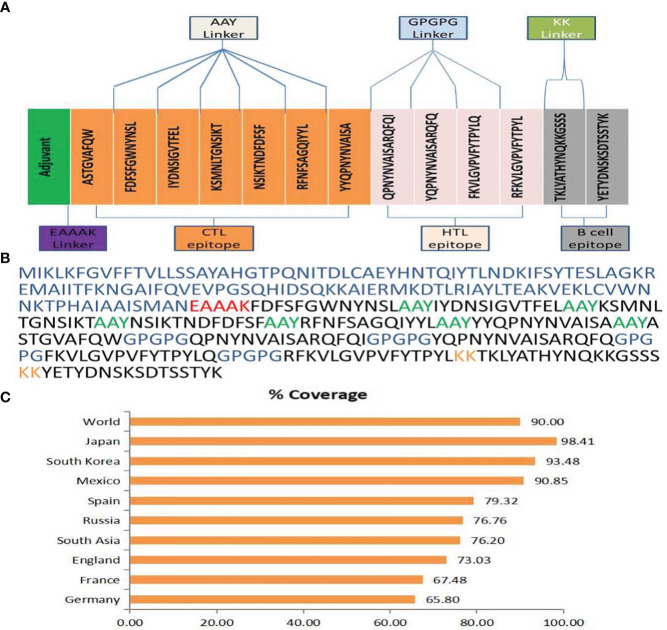
**(A)** Final MEV construct contain 344 amino acids followed by an adjuvant which is connected by EAAAK linker (purple color) and then followed by AAY connector (grey color) utilized to attach the CTL epitopes & GPGPG connector (cyan color) was utilized to fix the HTL epitopes merged through the KK linker (parrot color). **(B)** The multi-epitope vaccine construct sequence is color-coded: blue indicates the adjuvant, red represents the EAAAK linker, lime signifies the AAY linker, blue indicates the GPGPG linker, orange represents the KK linker, and black indicates CTL, HTL, and B-cell epitopes.**(C)** An overall Population coverage analysis of the selected T cell epitopes.

Population coverage is considered to be a significant parameter when it comes to MEV construction (88). At present, a total of 7 CTL and 4 HTL epitopes have been chosen to determine the population coverage, considering their respective alleles. Statistical analysis reveal that the shared coverage of 90.00% worldwide for chosen epitopes. The greatest population coverage was recorded in Japan that was 98.41%. While the reported population coverage for other countries were South Korea for 93.48%, Mexico for 90.85% and Spain for 79.32%. In short, this investigation validated that the filtered epitopes would prove to be potential candidate to construct MEV (**Figure 1**).

### Physiochemical and immunogenic profiling

When the produced vaccination’s homology was compared to the human proteome, no similarities were found. Our vaccine was found to be highly antigenic, non-allergenic, and non-toxic after its allergenicity, antigenicity, and toxicity were evaluated. ProtParam was utilized to assess the physiochemical properties (89). The resultant construct exhibited a molecular weight (MW) of 38,262.33 kDa and an isoelectric point (pI) of 9.37. The design of the vaccine showed an average half-life of thirty hours *in vitro*, more than twenty hours *in vivo* (Yeast), and more than ten hours *in vivo* (*E. coli*). The estimated GRAVY (grand average of hydropathy) was -0.271. Given all of these characteristics, *H. parainfluenzae* appears to be a suitable candidate for immunization.

### Secondary structure evaluation

To anticipate the secondary structure of the vaccine, the PSIPRED and SOPMA servers were deployed (90). The sequence analysis revealed that the alpha-helix comprised 25.58% of the sequence, consisting of 88 residues. Additionally, 30.52% of the sequence adopted extended chains with 105 residues, while 34.30% of the vaccine’s construct, encompassing 118 amino acids, exhibited a coil structure.

### Prediction of tertiary structure, refinement and validation

**Figure 2:**
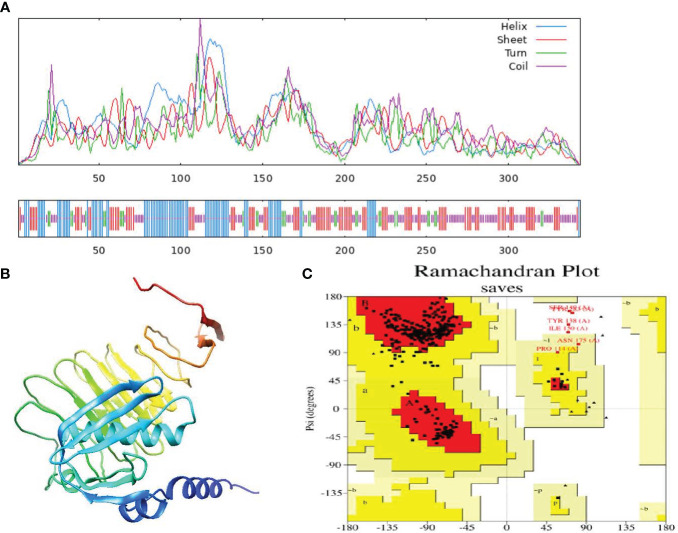
**(A)** Secondary structure prediction of the final multi-epitope vaccine construct by using SOPMA tool. Represent the helices (blue), sheets (red), coils (purple) and turn (green). The horizontal black bar at the bottom is representing the length of a protein. **(B)** The refined 3D structure of the vaccine construct. **(C)** Ramachandran Plot was generated to assess quality of the vaccine construct, revealing that 90.3% of amino acids residues in favored regions.

The web resource AlphaFold2 was used to estimate the tertiary structure of *H. parainfluenzae* (91). Furthermore, the Galaxy-refine server was employed to improve the structural refinement of the vaccine (50). Analysis of the improved model using the Ramachandran plot indicated that 90.3% of amino acids were situated in the favored regions, 8.3% were in the allowed regions, and only 1.3% were in the outlier regions, based on the enhanced model study (**Figure 2**). The improved model score found in the quality-check analysis of the ERRAT. These results show that the enhanced model has an exceptional 91.613 quality score (**Supplementary Figure 2**).

### Selection of B-cell epitopes

**Figure 3:**
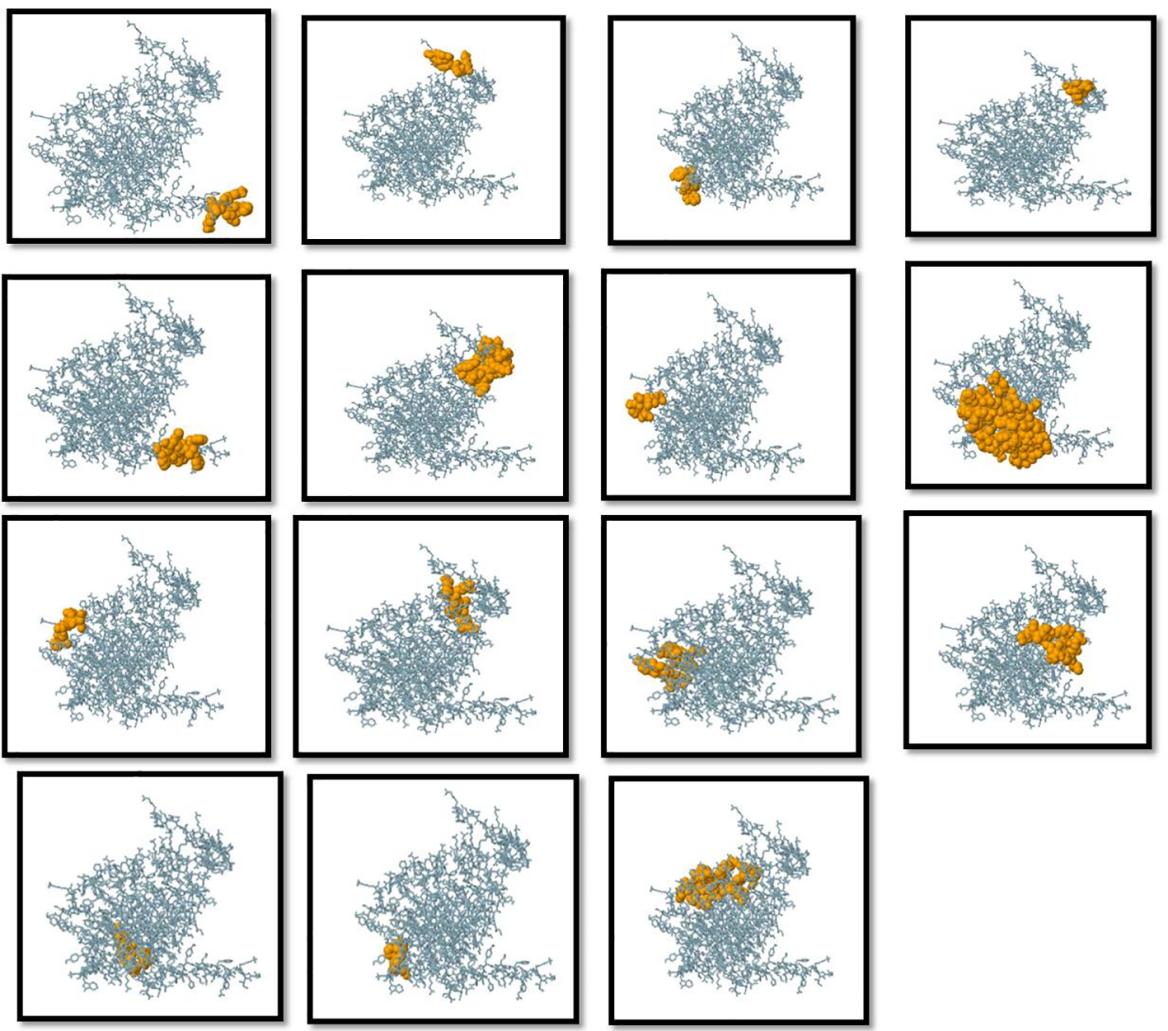
Three-dimensional representation of conformational or discontinuous B cell epitopes of the designed multi-epitope based vaccine. An orange surface represents the conformational or discontinuous B cell epitopes, and the bulk of the polyprotein is represented in grey sticks.

Humoral immunity is established through the production of antibodies by B-lymphocytes. Consequently, an effective vaccination should incorporate optimal B-cell epitope domains to stimulate the production of antibodies. The vaccine construct’s 15 conformationally-discontinuous epitopes of 3-47 residues (**Supplementary Table 2**). with score ranging 0.524-0.989 (as shown in **Figure 3**) and 12 linearly-continuous epitopes were predicted using ABCPred 2.0 with default parameters (**Supplementary Table 1**). The conformational B-cell epitopes were visualized during the vaccine production process using PyMOL v.1.3, a molecular graphics system (**Figure 3**).

### Disulfide engineering

Thirty-three amino acid residue pairings with the ability to create disulfide bonds were found by the DbD2 server. Subsequent evaluation based on chi3 and B-factor energy parameters narrowed down the selection to three specific pairs: PRO74-HIS78, GLY223-GLN245, and PHE246-ALA264. In each case, a cysteine residue was introduced to facilitate the formation of the disulfide bond. The evaluation process involved screening residues within the range of −87 to +97 chi3 values and ensuring an energy value of < 2.2. These criteria were employed to identify residues suitable for disulfide bond formation, enhancing the stability of the protein (**Supplementary Figure 1**).

### Molecular docking with host immune receptor

**Figure 4:**
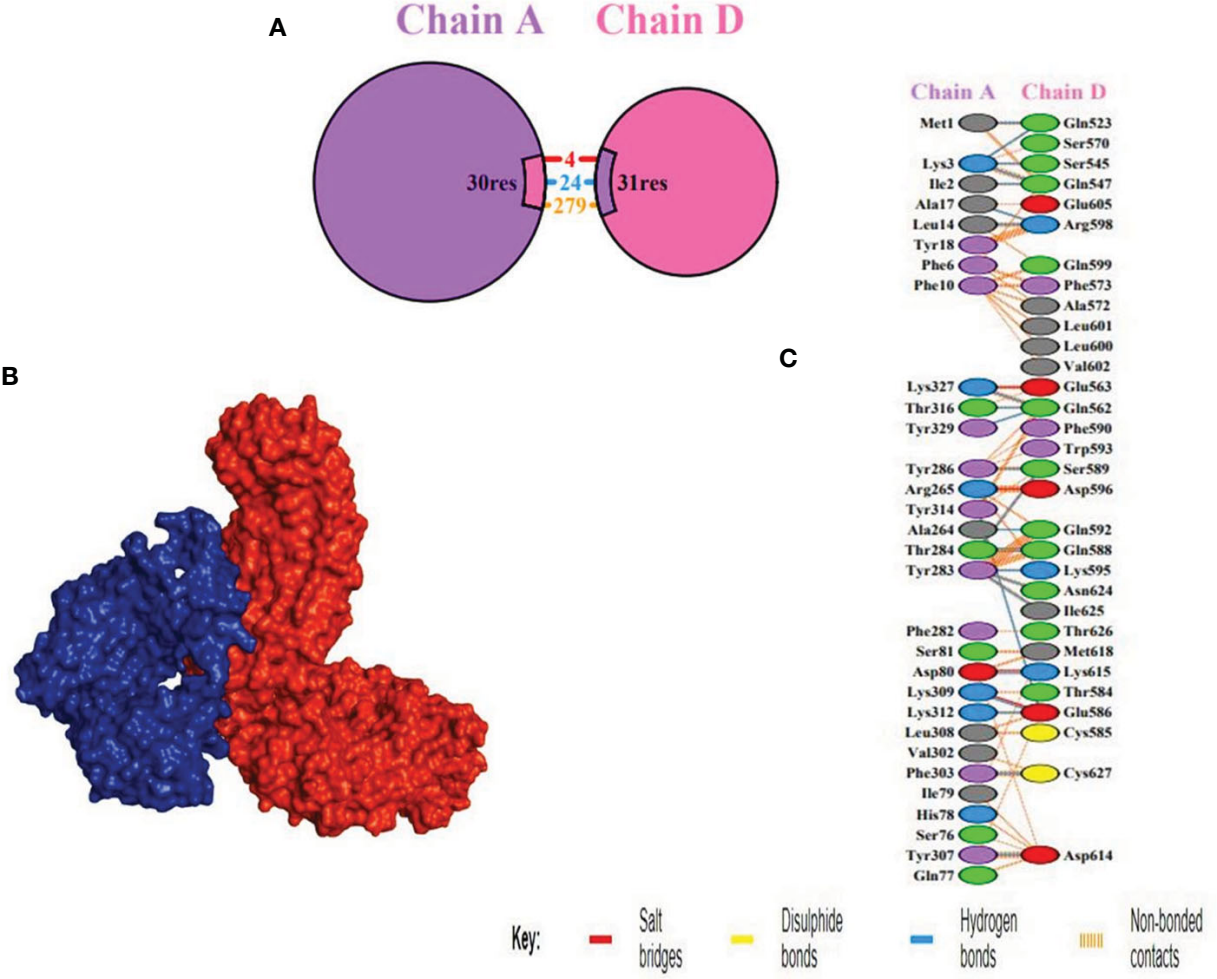
Docking between receptor, TLR4 and MEV **(A)** Chain A and D shown in purple and pink colors respectively, depicting the interacting residues. **(B)** Docking between receptor, chain D highlighted in blue, and MEV in red, showing the best binding affinity **(C)** Interacting residues illustration between vaccine construct and number of 24 hydrogen bonds were formed between the residues of the molecule and the residues of the vaccine.

TLR4 (PDB ID: 2z66) receptor was considered for docking, consisting of four chains. ClusPro was used to perform protein-protein docking between the vaccine design and TLR4 receptor to identify the optimal binding pose, assess stability, and determine binding affinity. Among ten models produced by ClusPro, the best docking complex was chosen by considering two points (1) the largest number of cluster and (2) possessing minimum energy. The first model fulfilled the criteria and had 87 members in cluster and -1098.4 kcal/mol energy, exhibiting the stability of the docking complex. Interaction between receptor and MEV was determined through PDB sum which unveiled that MEV showed better interaction with chain D of the receptor, formed 24 hydrogen bonds more than any other chain (**Table 5** and **Supplementary Table 3**). The thermodynamic parameters for the binding energy of the docking complex were obtained via the PRODIGY website. Specifically, the Gibbs free energy ΔG (ΔG = RT ln Kd) or equilibrium dissociation constant Kd was found to be 5.6x10-11 at 37oC and ΔG -14.o kcal/mol (**Figure 4**).

**Table 5:** Showing interaction detail between receptor and MEV.

Chain	No. of interface residues	Interface Area(A2)	No. of salt bridges	No. of Hydrogen bonds
A:C	13:13	509:526	1	9
B: D	13:13	524:508	1	8
B:E	7:4	206:283	2	5
D:E	28:22	1264:1262	1	26

### Normal mode analysis of MEV-receptor docked complex

**Figure 5:**
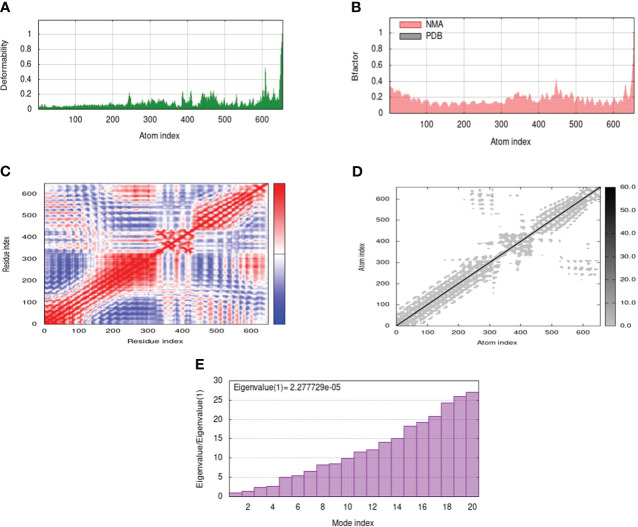
MD simulation of the docked complex of MEV, **(A)** deformability; **(B)** B-factor; **(C)** covariance index; **(D)** elastic network analysis; **(E)** eigenvalue.

Normal mode analysis between MEV and receptor docked complex was performed through iMODS which provided insights into deformability of residues. Deformability refers to how easily a protein can undergo any change in its three-dimensional structure. Peaks in the graph show high deformability indicating how easily protein three-dimensional structure can change. A comparison of the docked complex’s PDB and NMA can be seen in the B-factor graph. The graph’s greater peaks for NMA than PDB indicated that NMA data was expected to predict higher B-factors than PDB data. On the other hand, it clarifies that B-factor values found experimentally using PDB data were not as flexible or mobile as those indicated by computational simulations using NMA. The amount of energy needed to distort a structure is measured by its eigenvalue. The graph’s lower eigenvalues demonstrated that only a little amount of energy was required to distort the structure, confirming the molecular motion’s notable flexibility and stability. The variance graph has an inverse relation to the Eigenvalue graph. The red color indicates individual while the green color refers to cumulative variance. Relation between residues is indicated by the covariance matrix. The correlated, uncorrelated, and anti-correlated experience of amino acids is represented by red, white, and blue colors respectively. The docking complex showed a better correlation between pairs of residues which ensure the stability of dock complex (**Figure 5**).

### Immune simulation

**Figure 6:**
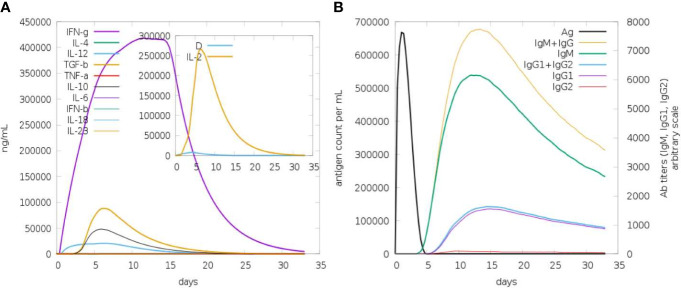
*In silico* immune response in which MEV serves as an antigen: **(A)** Immunoglobulins and B-cell isotypes formation when exposed to antigen; **(B)** cytokine and interleukin production in various stages with the Simson index.

Both primary and secondary immune responses are important when it comes to particular immune responses to a disease. IgG and IgM concentrations are significantly higher in the first reaction. The antigen is then reduced as a result of an increase in IgM, IgG1, IgG2, and IgG1 antibodies in both the secondary and primary stages. It is known that the cytokine and interleukin responses work quite well in this process. Reports show that the immunization produced an effective immune response, which resulted in the pathogen being eliminated on consecutive visits. These findings demonstrate the efficacy of the immune system in mounting a robust and adaptive response to combat the pathogen, ultimately contributing to an effective defense mechanism against the infection (**Figure 6**).

### *In silico* cloning

**Figure 7:**
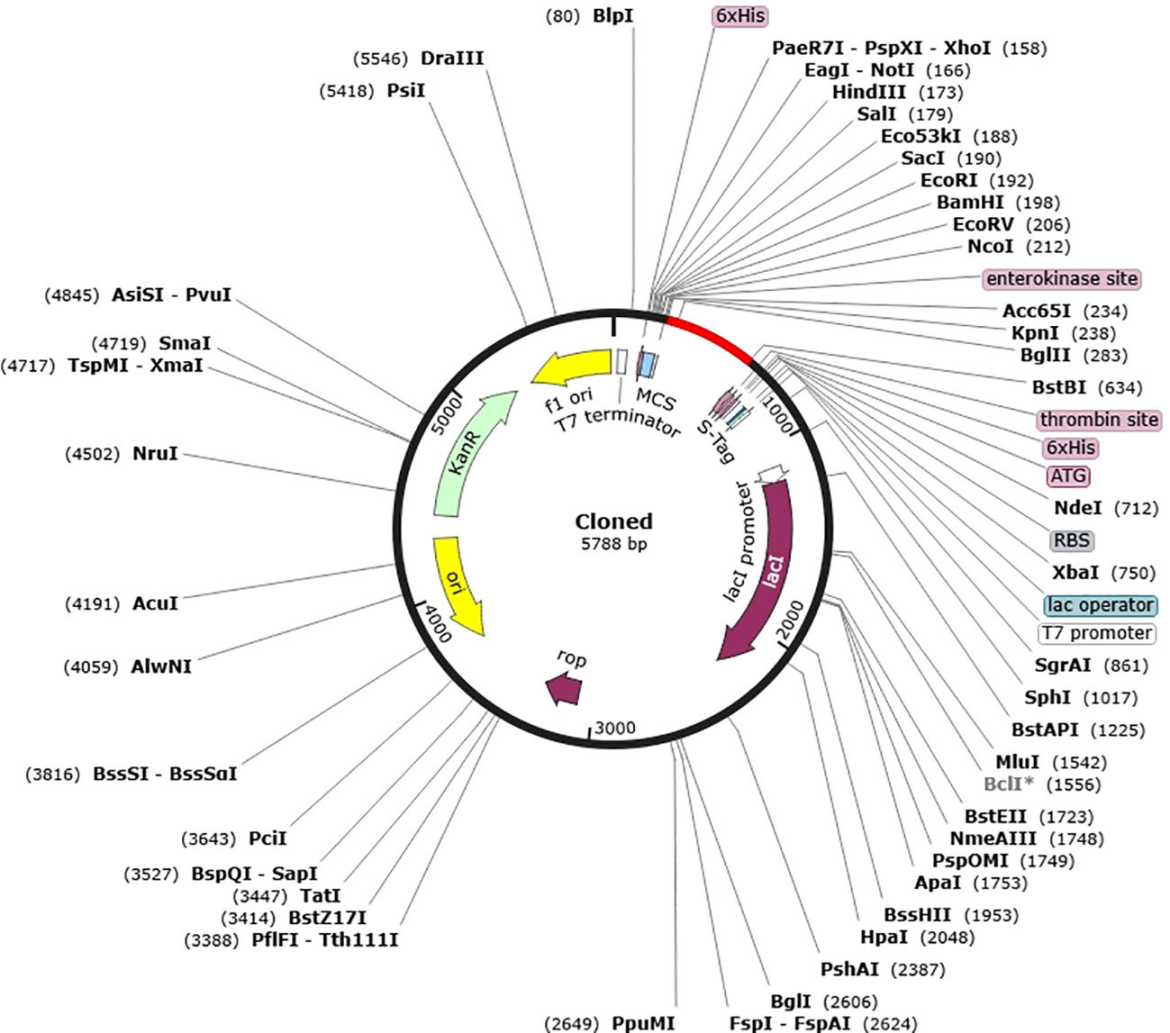
*In silico* cloning of vaccine into *E. coli* K12 host expression system. Plasmid is indicated in black however the introduced nucleotide sequence is in the red color.

To improve the vaccine protein’s expression efficiency in the *E. coli* host system, *in silico* cloning and codon optimization were carried out. The codons in the vaccination protein were modified to match the intended *E. coli* K12 host’s codon use. The Codon Adaptation Index (CAI) was calculated to be 0.9, indicating a high level of alignment, and the GC content of the optimized DNA was determined to be 49.32%.A CAI score of 1.0 was considered as an optimal compromise for efficient expression. The *E. coli* vector pET 30a (+) had the synthesized codon sequence carefully placed between the Nco1 and particular restriction sites. Furthermore, the resultant clone’s overall size of 5788 base pairs guaranteed that the expression vector’s optimized vaccination protein sequence would integrate successfully (**Figure 7**).

## Discussion

*Haemophilus parainfluenzae* is generally considered to be a commensal bacterium that inhabits the upper respiratory tract of humans, possess a significant threat to individuals with compromised immune systems or underlying medical conditions leading to opportunistic infections (92). Given its potential to cause serious health complications, the development of innovative therapeutic strategies is crucial. Immunoinformatics and reverse vaccinology emerge as effective approaches to identify novel vaccine candidates against deadly pathogens in time- and cost-effective manner. This integration of advanced technologies and computational tools has significantly transformed the landscape of vaccine development by streamlining the identification and analysis of potential antigens (93). The main aim of the current study was to develop a cutting-edge multi-epitope chimeric vaccine against *H. parainfluenzae* (70). The vaccine is intended to leverage both innate and adaptive immune responses, effectively boost the human immune system to provide protection against a wide range of diseases caused by this pathogen (94).

Our research primarily aimed to identify potential vaccine candidates for *H. parainfluenzae*, prioritizing two extracellular proteins (95). These selected proteins were characterized by their lack of homology to human proteins and have been reported to play essential role in the bacterium’s survival (96). It is anticipated to be found in the extracellular spaces, these proteins are expected to be among the initial molecules to interact with host cells (97). Consequently, these extracellular proteins were considered ideal candidates for vaccine development, as targeting them could potentially elicit a robust immune response against *H. parainfluenzae* (98). Among the proteins identified in our study, a key component to most Gram-negative bacteria is lipopolysaccharide (LPS). LPS plays pivotal roles in safeguarding bacteria from adverse environmental conditions and harmful substances, including antibiotics (99). Notably, LptD, a key player in LPS assembly, forms a distinctive 26-stranded β-barrel structure (100). To our knowledge, this specific β-barrel structure is the most comprehensively documented one to date (101). Understanding the structural characteristics of LptD sheds light on its functional significance in the context of LPS assembly and bacterial survival (100). The second protein under consideration is BamA, which belongs to the group of Outer Membrane (OM) β-barrel proteins (102). These proteins play vital roles in Gram-negative bacteria, as well as in mitochondria and chloroplasts, where they are involved in processes such as nutrient import, waste export, and signal transduction (103). The significance of BamA lies in its contribution to the structural integrity and functionality of the outer membrane, making it a crucial component for various cellular activities across different biological systems (104). The identification of CTL (Cytotoxic T Lymphocyte), HTL (Helper T Lymphocyte), and linear B-cell epitopes in our study adhered to stringent criteria (105). It takes B-cell epitopes to trigger humoral immune responses, leading to the neutralization of pathogenic agents and the establishment of a memory response for future encounters (106). However, T-cells, encompassing both CTLs and HTLs, play a crucial role in generating cellular immune responses that prevent the spread of disease by either eradicating infected cells or by releasing cytokines with anti-microbial properties, guaranteeing protection that can last for decades (107). Additional prioritization based on antigenicity, non-allergenicity, and non-toxicity was used in the selection of B- and T-cell epitopes (26). Interestingly, the selected epitopes covered around 90% of the world’s population combined. We used overlapping lead MHC-I, MHC-II, and B-cell epitopes to develop multi-epitope-based chimeric vaccine constructs, incorporating particular linker and adjuvant sequences for increased efficacy (105). Similar in-silico techniques have been effectively used to create vaccine constructions that protect against a variety of diseases (70). Notably, this approach has been applied to pathogens such as *Salmonella Typhimurium*, *Trypanosoma vivax*, *Acinetobacter baumannii*, COVID-19, *Ebola virus*, and *Marburg virus*. Importantly, these *in-silico* designed vaccine constructs have undergone experimental validation, confirming their potential efficacy in immunological responses (108). It is expected that the vaccination models developed in this work will exhibit advantageous properties, such as strong antigenicity combined with minimal allergenicity and toxicity (109). Predicted physicochemical characteristics indicate that the vaccine designs have increased hydrophilicity and stability (70). These characteristics suggest that they may stimulate strong immunogenic reactions in the human immune system (16). The models also show good solubility, low molecular weight, and thermodynamic stability, suggesting that the vaccine constructions might be produced and administered in the host in an effective manner (110).

To evaluate the effectiveness of the vaccination, it is imperative to understand the biomolecular interactions between the human immune cell receptor molecules and the proposed vaccine. This necessitates the acquisition of three-dimensional structural data (111). In this study, a diverse array of computational tools was utilized to conduct validations and predict the tertiary structures of the suggested vaccinations (70). The Ramachandran plot values provided evidence of substantial improvement and desired characteristics in the quality and stability of the resulting vaccine constructs (76). This validation process ensures that the proposed vaccines possess structural attributes conducive to effective interactions with the immune system’s cellular components (29). Previous research has indicated that human Toll-like receptors (TLRs) play a crucial role in recognizing pathogenic peptides and initiating immune responses against specific pathogens (63). To evaluate the potential effectiveness of *H. parainfluenzae* vaccines, molecular docking analyses were performed, specifically focusing on their interactions with human TLR4 receptors (112). This analysis aims to provide insights into the molecular interactions between the proposed vaccines and TLR4, shedding light on the potential for stimulating immune responses against *H. parainfluenzae* (113). Repeated exposure to the antigenic vaccine construct resulted in the activation of stronger immune responses. This included the stimulation of Helper T cells and the development of memory B and T cells. The enhanced production of Immunoglobulins (Ig) and activation of T-helper cells contributed to a robust humoral immune response (114). A comparable study observed similar immune patterns in response to various pathogens. Furthermore, predictions made by the C-ImmSim application were experimentally validated in recent studies (115). These studies reported immunization patterns against bacterial antigens that closely aligned with the predictions made by the C-ImmSim resource (116). This validation supports the reliability of the predictions and underscores the consistency of the observed immune responses in experimental settings (117). The findings suggest that the prioritized vaccine construct holds substantial potential to engage human Toll-like receptors (TLR) and trigger both humoral and cell-mediated immune responses against *H. parainfluenzae* (118). Through computational restriction cloning of the vaccine cDNA sequence into an *E. coli* plasmid, the expression of the vaccine construct in a bacterial expression system is insured (119). Collectively, these analyses establish a foundation for the development of an effective anti-*H. parainfluenzae* vaccine, demonstrating its capability to elicit robust immunological responses within the human host immune system (120).

The present study, introduces a multi-epitope chimeric vaccine design incorporating the LptD and BamA protein components as an approach to address antigenic complexities (121). However, it is important to acknowledge certain limitations in the current study. The construction of immune-informatics-based vaccines heavily relies on prediction methods, introducing uncertainties regarding the level of protection against LptD and BamA infections (122). The accuracy of these prediction approaches may be constrained by standard benchmarking, restricted prediction methodologies, and a lack of precise datasets for diverse computational studies (123). While immune-informatics predictions have shown positive outcomes in some recent case reports, the findings of this study necessitate validation through *in vitro* and *in vivo* bioassays to ascertain the safety and efficacy of the proposed vaccine against *H. parainfluenzae* (124).

## Data availability statement

The original contributions presented in the study are included in the article/**Supplementary Material**. Further inquiries can be directed to the corresponding author.

## Author contributions

SA: Conceptualization, Investigation, Methodology, Project administration, Software, Writing – original draft, Writing – review & editing. HT: Conceptualization, Data curation, Formal analysis, Validation, Visualization, Writing – review & editing. SM: Conceptualization, Formal analysis, Methodology, Visualization, Writing – review & editing. MS: Conceptualization, Data curation, Formal analysis, Project administration, Writing – review & editing. TN: Funding acquisition, Writing – review & editing, Conceptualization, Data curation, Validation. MF: Formal analysis, Writing – review & editing, Validation. TA: Conceptualization, Formal analysis, Funding acquisition, Resources, Writing – review & editing. AA: Funding acquisition, Methodology, Writing – review & editing, Investigation, Project administration, Software, Visualization. NA: Conceptualization, Formal analysis, Writing – review & editing, Funding acquisition, Methodology, Resources. IM: Conceptualization, Formal analysis, Supervision, Validation, Writing – review & editing.

## Supplementary material

Supplementary Figure 1 Stability of the vaccine construct by disulfide bond engineering. Three pairs of amino acids represented in the pair energy shown in the sphere form in the red, orange and blue color.

Supplementary Figure 2 ERRAT score of vaccine construct.

Supplementary Table 1 Linear B-cell epitopes in the vaccine construct.

Supplementary Table 2 ElliPro server predicted a total of 187 residues, located in four discontinuous B-cell epitopes.

Supplementary Table 3 MEV receptor docked complex amino acids detail in tabular form.
