# Supplementary figures and images for: Designing of a multi-epitopes based vaccine against Haemophilius parainfluenzae and its validation through integrated computational approaches
Source: Front Immunol. 2024 Apr 16;15:1380732. doi: 10.3389/fimmu.2024.1380732 (PMC11058264; doi:10.3389/fimmu.2024.1380732)

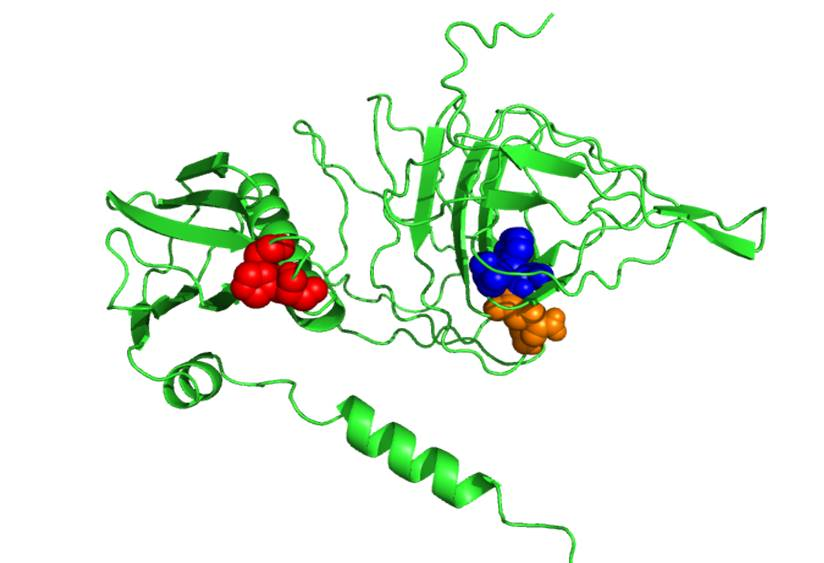

Supplement: Supplementary Figure 1 — Stability of the vaccine construct by disulfide bond engineering. Three pairs of amino acids represented in the pair energy shown in the sphere form in the red, orange and blue color. [file Image_1.tiff]

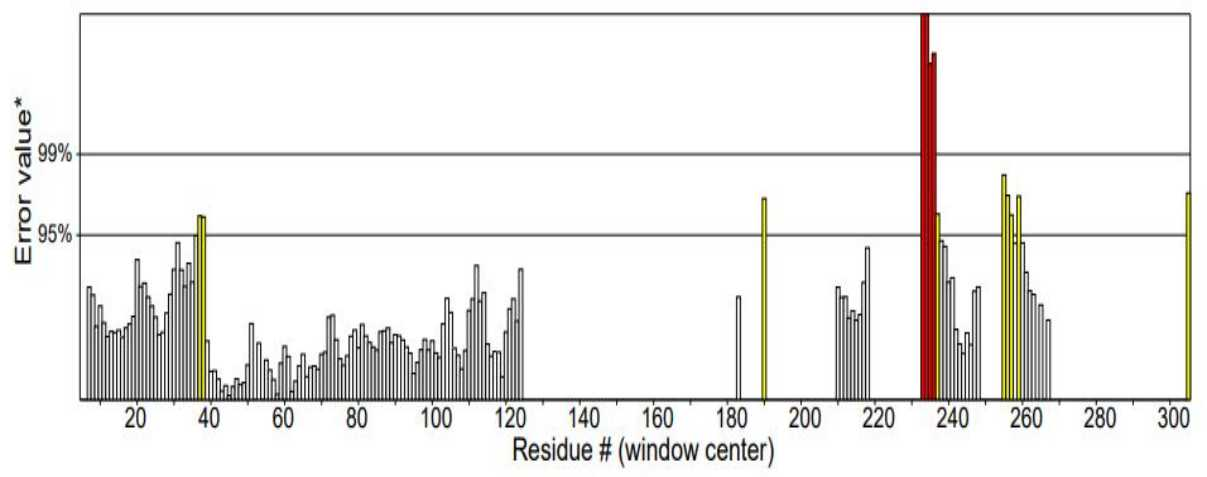

Supplement: Supplementary Figure 2 — ERRAT score of vaccine construct. [file Image_2.tiff]
